# *SLC26A1* is a major determinant of sulfate homeostasis in humans

**DOI:** 10.1172/JCI161849

**Published:** 2023-02-01

**Authors:** Anja Pfau, Karen I. López-Cayuqueo, Nora Scherer, Matthias Wuttke, Annekatrin Wernstedt, Daniela González Fassrainer, Desiree E.C. Smith, Jiddeke M. van de Kamp, Katharina Ziegeler, Kai-Uwe Eckardt, Friedrich C. Luft, Peter S. Aronson, Anna Köttgen, Thomas J. Jentsch, Felix Knauf

**Affiliations:** 1 Department of Nephrology and Medical Intensive Care, Charité-Universitätsmedizin Berlin, Berlin, Germany.; 2 Leibniz-Forschungsinstitut für Molekulare Pharmakologie (FMP) and Max-Delbrück-Centrum für Molekulare Medizin (MDC), Berlin, Germany.; 3 Institute of Genetic Epidemiology, Faculty of Medicine and Medical Center and; 4 Spemann Graduate School of Biology and Medicine (SGBM), University of Freiburg, Freiburg, Germany.; 5 SYNLAB MVZ Humane Genetik, Munich, Germany.; 6 Metabolic Laboratory, Department of Clinical Chemistry, Amsterdam Neuroscience and; 7 Department of Human Genetics, Amsterdam UMC, Vrije Universiteit Amsterdam, Amsterdam, Netherlands.; 8 Department of Radiology, Charité-Universitätsmedizin Berlin, Berlin, Germany.; 9 Department of Nephrology and Hypertension, Friedrich-Alexander-Universität Erlangen-Nürnberg, Erlangen, Germany.; 10 Department of Internal Medicine, Section of Nephrology, Yale University School of Medicine, New Haven, Connecticut, USA.; 11 CIBSS – Centre for Integrative Biological Signalling Studies, Albert-Ludwigs-University Freiburg, Freiburg, Germany.; 12 NeuroCure Cluster of Excellence, Charité-Universitätsmedizin Berlin, Berlin, Germany.

**Keywords:** Genetics, Nephrology, Cartilage, Epithelial transport of ions and water, Genetic diseases

## Abstract

Sulfate plays a pivotal role in numerous physiological processes in the human body, including bone and cartilage health. A role of the anion transporter SLC26A1 (Sat1) for sulfate reabsorption in the kidney is supported by the observation of hyposulfatemia and hypersulfaturia in *Slc26a1*-knockout mice. The impact of *SLC26A1* on sulfate homeostasis in humans remains to be defined. By combining clinical genetics, functional expression assays, and population exome analysis, we identify SLC26A1 as a sulfate transporter in humans and experimentally validate several loss-of-function alleles. Whole-exome sequencing from a patient presenting with painful perichondritis, hyposulfatemia, and renal sulfate wasting revealed a homozygous mutation in *SLC26A1*, which has not been previously described to the best of our knowledge. Whole-exome data analysis of more than 5,000 individuals confirmed that rare, putatively damaging *SCL26A1* variants were significantly associated with lower plasma sulfate at the population level. Functional expression assays confirmed a substantial reduction in sulfate transport for the *SLC26A1* mutation of our patient, which we consider to be novel, as well as for the additional variants detected in the population study. In conclusion, combined evidence from 3 complementary approaches supports SLC26A1 activity as a major determinant of sulfate homeostasis in humans. In view of recent evidence linking sulfate homeostasis with back pain and intervertebral disc disorder, our study identifies SLC26A1 as a potential target for modulation of musculoskeletal health.

## Graphical abstract

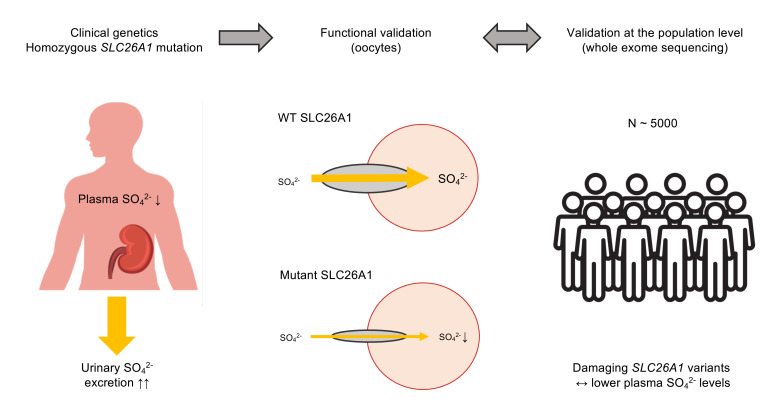

## Introduction

Sulfate is involved in numerous metabolic processes in humans. However, it is not measured as part of routine clinical chemistry tests, and few specialized laboratories provide this service. Therefore, clinical data related to sulfate disturbances are necessarily sparse (1, 2). The kidney plays a major role in maintaining plasma sulfate at its physiological concentration of approximately 300 μM (2, 3). Sulfate is freely filtered in the kidney glomerulus and then reabsorbed across the proximal tubule epithelial cell. Transcellular sulfate reabsorption is mediated by the apical membrane sodium-sulfate cotransporter SLC13A1 working in series with the basolaterally located SLC26A1 (sulfate anion transporter 1, Sat1) (3, 4).

Disturbances in sulfate homeostasis have been described to cause osteoarthritis and chondropathies (1, 5–8). For instance, a recessively inherited form of osteochondrodysplasia in dogs has been demonstrated to be due to hyposulfatemia as a consequence of a mutation in *SLC13A1* (8). Moreover, a recent genome-wide association study demonstrated that intervertebral disc disorders, a very common example of painful cartilage disease, are also associated with low plasma sulfate concentrations and rare loss-of-function variants of *SLC13A1* (7).

In contrast, the pathophysiological role of genetic variation in *SLC26A1* in human sulfate homeostasis is less clear; SLC26A1 is a pH-sensitive anion exchanger for sulfate and bicarbonate, which also mediates oxalate transport (3, 9). In *Slc26a1*-knockout mice, hyposulfatemia and hypersulfaturia have been reported (3, 10, 11), whereas the impact of *Slc26a1* on oxalate homeostasis leading to hyperoxaluria and urolithiasis remains controversial (10, 12, 13). Biallelic mutations of *SLC26A1* were described in 2 patients with calcium oxalate nephrolithiasis (14), but the impact on sulfate balance was not assessed. The present work aims to elucidate the role of *SLC26A1* in human sulfate homeostasis by genetic analysis of a familial case and functional analyses, as well as at the population level in a large cohort of almost 5,000 participants.

## Results

### Detection of a homozygous SLC26A1 mutation in a patient with hyposulfatemia and renal sulfate wasting.

**Figure 1:**
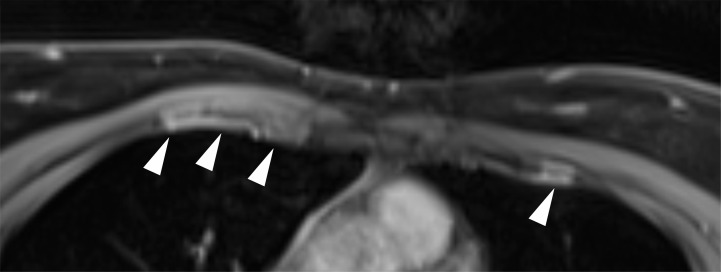
MRI demonstrated perichondritis at the level of the third and fourth ribs. Axial fat-saturated T1-weighted images after administration of intravenous contrast agent (gadolinium): Linear contrast enhancement of the costal cartilage on both sides at the level of the third and fourth ribs (white arrowheads), predominantly at the costochondral junction, consistent with perichondritis. Note also the subtle diffuse enhancement of the adjacent soft tissue on the right side.

**Figure 2:**
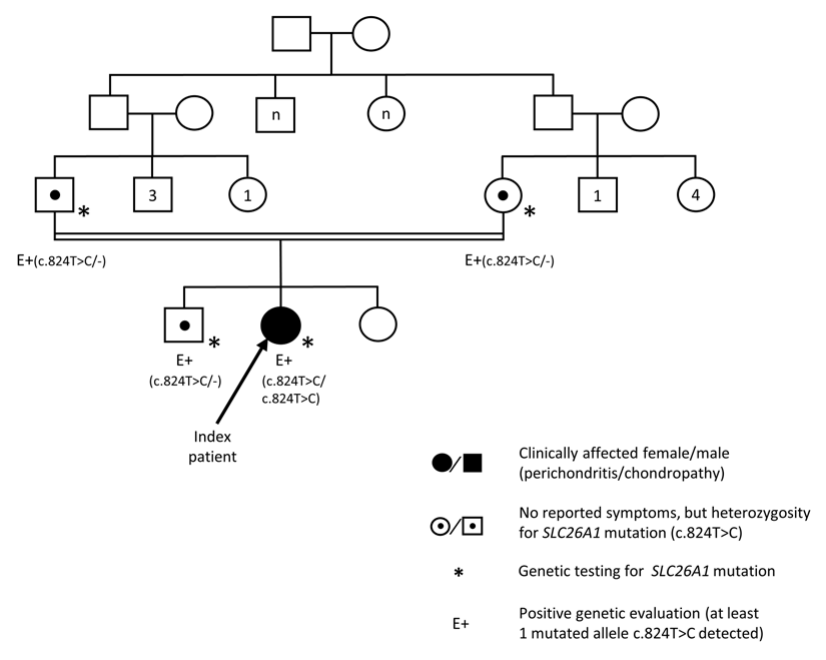
Clinical workup revealed a family history for consanguinity. The parents of the index patient were first cousins. Genetic testing was only available in 4 family members (patient, parents, and 1 sibling). Filled symbols indicate a clinically affected individual (chondropathy). Symbols with a central dot represent an individual with no reported symptoms, but who was heterozygous for *SLC26A1* mutation in genetic testing. E+ marks individuals in whom at least 1 mutated allele (c.824T>C) was detected.

A 33-year-old patient presenting at our clinic had been suffering from intermittent chest pain radiating from her costochondral joints that defied diagnosis for more than 6 years. Magnetic resonance imaging (MRI) that had been performed at the onset of the symptoms had revealed changes consistent with perichondritis of the third and fourth right costovertebral joints, with lesser findings on the left side (Figure 1). Despite repeated consultation of numerous physicians representing various specialties, including an extensive rheumatological workup performed at 3 different academic medical centers, the perichondritis remained unexplained. An additional finding of note was a kidney stone of 13 mm diameter in an upper calyx of the right kidney. Her kidney function was normal. Her family tree revealed that her parents were first cousins (Figure 2). Her 2 siblings did not show similar symptoms. In view of the unresolved clinical presentation and the consanguinity of the parents, comprehensive genetic testing was performed.

**Figure 3:**
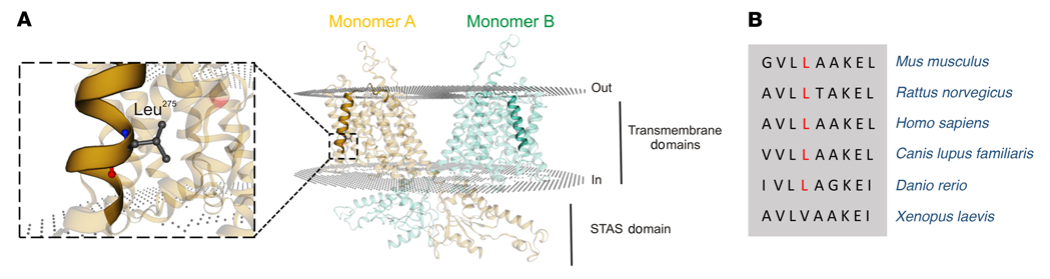
Mapping of SLC26A1 variant on a homology model. (**A**) Based on the structure of Prestin EMD-23334 (43), a SLC26A1 homology model was constructed by the Swiss model server. The SLC26 family transporters are dimers; each monomer is indicated in a different color. The fourth transmembrane domain is highlighted, illustrating the position of Leu^275^. STAS domain, sulfate transporter and anti–sigma factor antagonist domain. (**B**) Partial alignment of SLC26A1 among different species points to a high level of conservation.

Whole-exome sequencing, as confirmed by Sanger sequencing, revealed that the patient was homozygous for a mutation (c.824T>C, p.Leu275Pro) in the anion transporter 1 (*SLC26A1*). Amino acid Leu^275^ is located in the fourth transmembrane domain of SLC26A1 and was mutated to proline, a helix-breaking residue. The identified missense change affected a residue highly conserved among different species (Figure 3, A and B). In silico predictive tools consistently classified the *SLC26A1* variant as damaging/deleterious (SIFT score < 0.05, PolyPhen-2 score = 0.987) (15). To our knowledge, this mutation has not been described previously. The asymptomatic parents and her brother were heterozygous; the remaining sister chose not to be tested.

As discussed above, SLC26A1 is an anion exchanger capable of mediating both sulfate and oxalate transport when expressed in heterologous expression systems (3, 11). As shown in Table 1 and Supplemental Table 1 (supplemental material available online with this article; https://doi.org/10.1172/JCI161849DS1), our patient had markedly lower plasma sulfate compared with controls, reference values reported in the literature, and the mother, a heterozygous carrier of p.Leu275Pro, who had low-normal sulfate levels (2, 16–18). The fractional excretion index (FEI) (16) of sulfate in the urine was 0.24 despite the presence of hyposulfatemia. The FEI of sulfate is reported to be 0.17–0.34 under normal conditions (16), but it is known that sulfate excretion falls to very low levels of around 10% or less when sulfate depletion is induced by restriction of dietary sulfate (17, 19, 20), or in states of increased sulfate demand such as pregnancy (18) even when plasma sulfate concentrations are normal. Thus, the observation that the patient had a mean FEI of sulfate of 24% when plasma sulfate was severely depressed indicated urinary sulfate wasting with inappropriately high fractional sulfate excretion in the urine. Twenty-four-hour urine collections for oxalate excretion revealed urinary oxalate values between 21.8 and 48.6 mg/day (mean 33.7 mg/day), i.e., normal to only mildly elevated (Table 1) (21). Together, these observations indicate that the p.Leu275Pro mutation clearly affected plasma sulfate levels due to urinary sulfate wasting, with minor, if any, impact on urinary oxalate levels.

### Functional analyses of the detected SLC26A1 mutation (p.Leu275Pro) in Xenopus laevis oocytes confirm reduced sulfate transport.

**Figure 4:**
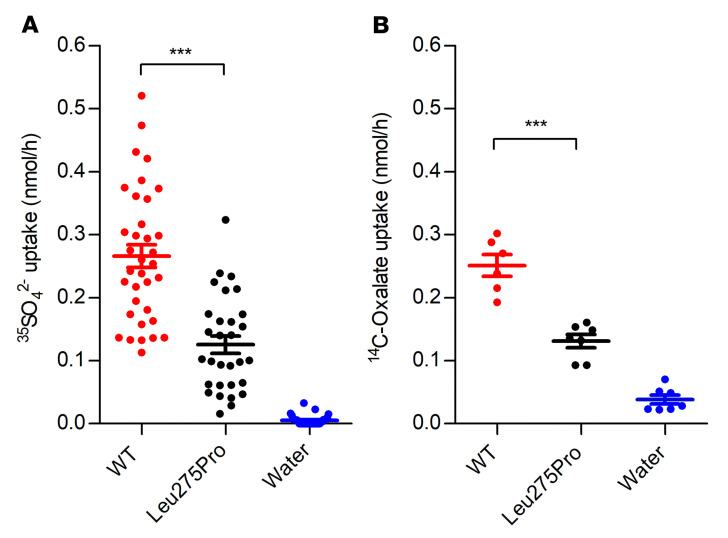
Reduced sulfate and oxalate transport in *Xenopus laevis* oocytes expressing mutant SLC26A1 (p.Leu275Pro). (**A**) SLC26A1-mediated SO_4_^2−^ and (**B**) oxalate uptake by oocytes previously water injected or injected with 10 ng of cRNA encoding WT *SLC26A1* or Leu275Pro mutant *SLC26A1*. Uptake was carried out from a bath solution containing 1 mM SO_4_^2−^ or 1 mM oxalate for 15 minutes. The SO_4_^2−^ uptake experiments were performed with oocytes from 4 different frogs (and 3 different cRNA preparations) with a total number of 35 WT, 30 Leu275Pro, and 33 water-injected oocytes, and for oxalate, 6 WT, 7 Leu275Pro, and 7 water-injected oocytes. Data are presented as mean ± SEM. ***P < 0.001 by 1-way ANOVA with Bonferroni’s multiple-comparison test.

**Figure 5:**
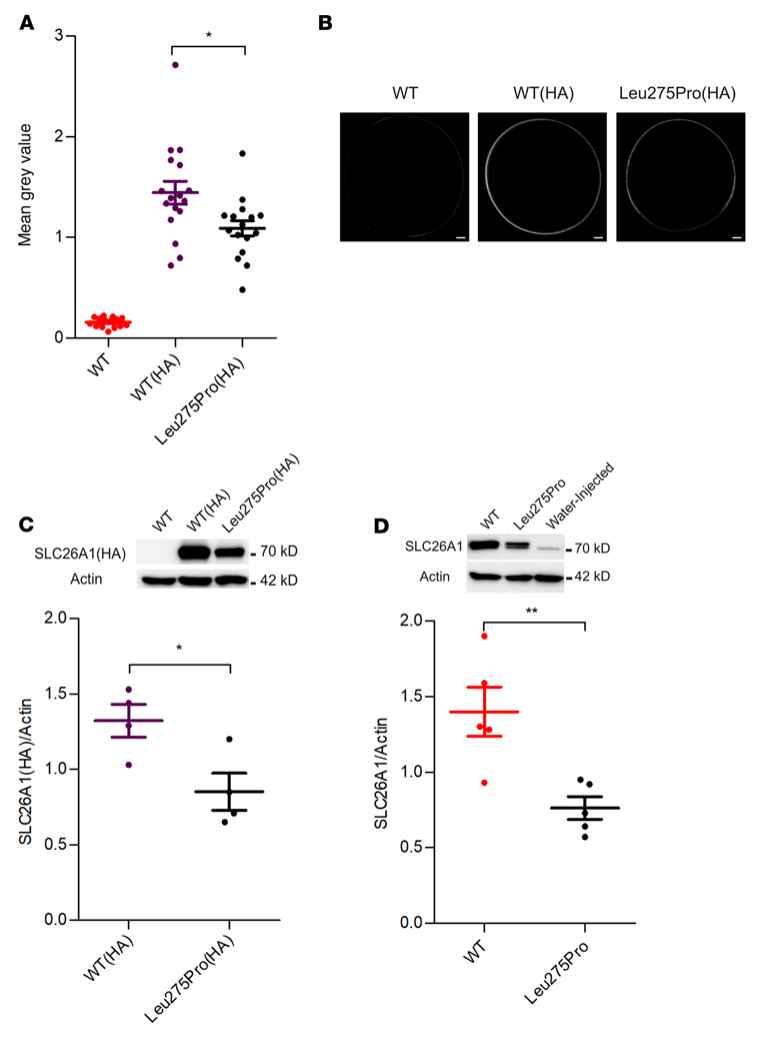
Reduced cell surface expression of mutant SLC26A1 (p.Leu275Pro) in *Xenopus laevis* oocytes. (**A**) Quantification of the mean gray value intensity of immunofluorescence in nonpermeabilized oocytes, carrying HA as a tag on the second extracellular loop of WT *SLC26A1* [WT(HA)] or mutant *SLC26A1* [Leu275Pro(HA)]. Oocytes injected with *SLC26A1* without HA were used as a control (WT); 10 ng of the respective cRNAs was injected. (**B**) Representative oocyte immunofluorescence pictures. Scale bars: 100 μm. WT *n* = 15, WT(HA) *n* = 17, and Leu275Pro(HA) *n* = 16; oocytes were obtained from 3 different frogs (3 different cRNA preparations). (**C** and **D**) Quantification of SLC26A1 protein expression by immunoblots of membranes from oocytes expressing HA epitope–tagged (**C**) or untagged (**D**) WT or Leu275Pro mutant *SLC26A1*. An antibody against the HA tag was used in **C**, and an anti-SLC26A1 antibody (C-terminus) in **D**. Western blots include lanes from WT SLC26A1 or water-injected oocytes as specificity controls. For these experiments, 10–15 oocytes injected with 10 ng of the corresponding cRNA were pooled and actin was used as a loading control. Western blots were repeated at least 3 times with oocytes from 3 different frogs (3 different cRNA preparations were used). *P < 0.05; **P < 0.01 by 1-way ANOVA with Bonferroni’s multiple-comparison test (**A**) or unpaired, 2-tailed *t* test (**C** and **D**).

To confirm the functional relevance of the *SLC26A1* mutation (p.Leu275Pro), we expressed wild-type (WT) *SLC26A1* and the mutant detected in our patient in *Xenopus laevis* oocytes. The oocytes expressing mutant transporter exhibited greatly reduced sulfate and oxalate transport, compared with the WT protein (Figure 4, A and B). To determine the expression of SLC26A1 on the cell surface, an HA tag was inserted into the second extracellular loop of the WT SLC26A1 and the Leu275Pro mutant (Supplemental Figure 1). There was a marked reduction in cell surface expression of the mutant transporter compared with WT, as indicated by immunofluorescence studies performed in nonpermeabilized oocytes (Figure 5, A and B). Western blotting performed on membrane proteins of oocytes expressing WT and mutant SLC26A1, using antibodies against the HA tag or the native SLC26A1 protein, revealed a decrease in overall SLC26A1 expression levels of similar magnitude (Figure 5, C and D, respectively). These findings suggest that a decrease in SLC26A1 protein levels, possibly by increased degradation of partially misfolded transporter proteins, importantly contributes to the reduced plasma membrane transport activity of the mutant. These studies therefore verify that p.Leu275Pro is a human loss-of-function mutation.

### Rare SLC26A1 variants also impact plasma sulfate levels at the population level.

**Figure 6:**
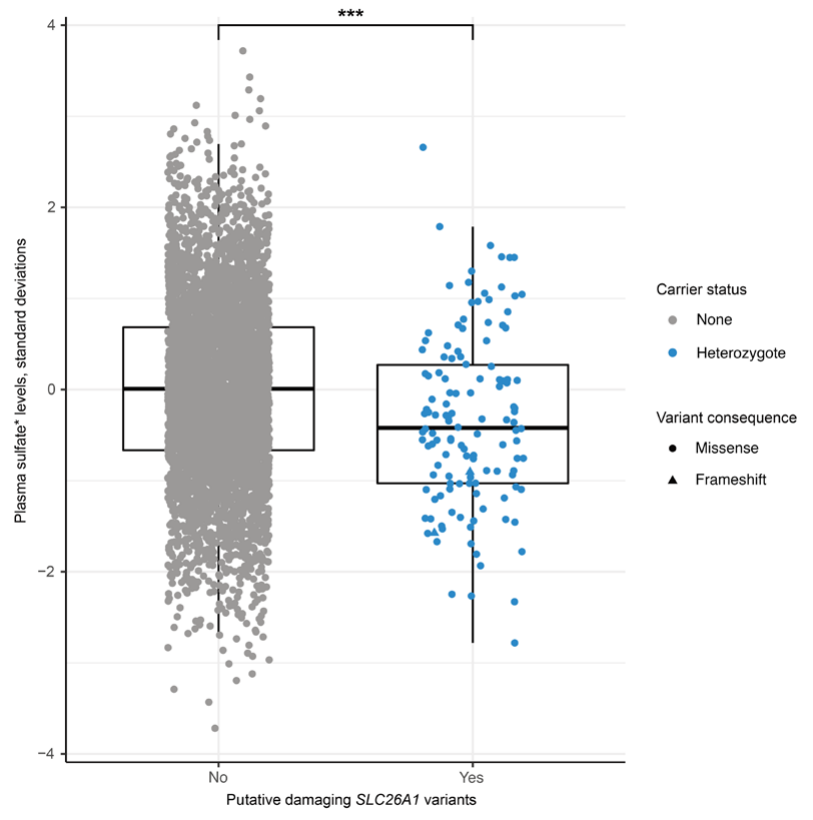
Carriers of putative damaging *SLC26A1* variants in the GCKD study have lower median plasma sulfate levels than noncarriers. Plasma sulfate levels (*y* axis) displayed by *SLC26A1* rare variant carrier status (*x* axis). The *y* axis represents sulfate levels after inverse normal transformation, with units corresponding to standard deviations. *Denotes that Metabolon is highly confident in metabolite identity but a standard for this metabolite has not been run. The symbol color indicates observed rare variant carrier status, and symbol shape variant consequence (triangle, frameshift; circle, missense). The boxes range from the 25th to the 75th percentile of sulfate levels, the median is indicated by a line, and whiskers end at the last observed value within 1.5 × (interquartile range) away from the box. ***P < 0.001 (*P* value from aggregate variant test = 3.01 × 10^–5^). Metabolon measurements yield semiquantitative rather than absolute metabolite levels.

Clinical genetics studies of single patients usually require confirmation in at least one other unrelated patient or family. Recent advances in population-level whole-exome sequencing now offer an opportunity for orthogonal validation of genetic findings through complementary population-level analysis. Therefore, to investigate whether SLC26A1 activity is a major determinant of sulfate homeostasis in humans, we searched for associations between rare, coding *SLC26A1* variants that were predicted to be damaging (see Supplemental Table 2) and plasma sulfate levels among 4,708 participants of the prospective German Chronic Kidney Disease (GCKD) study that enrolled a total of 5,217 patients with moderate chronic kidney disease (22). We detected 43 such rare, coding variants (alleles) among 130 different participants (hereinafter referred to as carriers). The 130 carriers had significantly lower average levels of plasma sulfate compared with 4,578 GCKD participants not carrying any such variant (*P* = 3.01 × 10^–5^, gene-burden test) (Figure 6 and Supplemental Table 2). All variant carriers were heterozygous. As a negative control, we also repeated the gene-burden test and included only synonymous variants in *SLC26A1*, which are presumed to be neutral (23), and found no significant association with plasma sulfate (*P* = 0.7), as expected.

**Figure 7:**
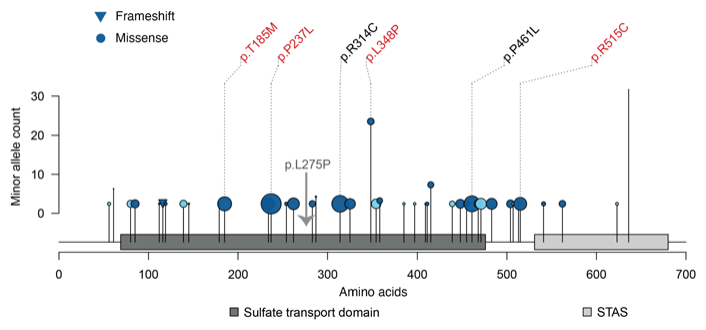
Qualifying *SLC26A1* variants in the GCKD study and their localization, frequency, consequence, effect size, and effect direction. All qualifying rare, coding variants included in the aggregate variant test are plotted at the corresponding amino acid position of *SLC26A1* (Uniprot ID Q9H2B4) on the *x* axis. The protein domains are based on Pfam 35.0. The *y* axis represents the minor allele count of each variant among the 4,708 GCKD study participants. The shape of the variant’s lollipop corresponds to its predicted consequence. The position of our patient’s mutation (Leu275Pro) is noted as well. The size of the variant’s lollipop represents the absolute value of the effect size of a single variant test with inverse normal transformed plasma sulfate levels (Supplemental Table 2), and the color indicates the direction of the effect size: the darker blue represents negative effect sizes and the lighter blue positive ones. All variants with an effect size of less than –1 or with a *P* value of less than 0.05 are labeled with their predicted amino acid exchange, where the ones with a *P* value of less than 0.05 are labeled in red. STAS domain, sulfate transporter and anti–sigma factor antagonist domain.

Figure 7 shows that the detected potentially damaging 43 variants were distributed across the entire coding region. Variants with the largest negative effects on plasma sulfate such as p.Pro237Leu, p.Arg314Cys, and p.Pro461Leu mapped into the sulfate transport domain of *SLC26A1*, consistent with a loss-of-function mechanism. Sequential omission of identified variants from testing their aggregate effect on sulfate levels supported the presence of several independent contributing variants (Supplemental Figure 2). All 43 variants, their frequencies, predicted consequences, effect sizes, and *P* values for their individual association with plasma sulfate are listed in Supplemental Table 2. They represent a valuable resource both for further experimental studies and when additional patients with unexplained hyposulfatemia, who may be homozygous or compound heterozygous for any of the variants, are identified in the future.

Since all participants of the GCKD study had chronic kidney disease, we assessed whether their kidney filtration function, quantified as estimated glomerular filtration rate (eGFR), influenced the variants’ association with plasma sulfate levels. As shown in Supplemental Figure 3, the 30 carriers of potentially damaging *SLC26A1* variants who had an eGFR of 60 mL/min/1.73 m^2^ or greater, i.e., better kidney function, had on average even lower plasma sulfate levels than those with reduced eGFR (<60 mL/min/1.73 m^2^) (*P* < 0.001, unpaired *t* test). This observation was corroborated when repeating the subanalysis for only carriers of the same variant (namely Asp636Tyr and Leu348Pro, for which a sufficient number of carriers was available). Thus, the effect of the detected *SLC26A1* variants on plasma sulfate levels can also be expected to be detected in persons without reduced kidney function.

In the GCKD cohort, no association could be found between carrier status and self-reported kidney stones at the 6-year visit, when this information was collected (Fisher’s exact test, *P* > 0.1).

### Functional analyses reveal reduced sulfate transport for SLC26A1 variants identified in the GCKD study.

To test whether the potentially damaging *SLC26A1* variants identified in the GCKD study impair sulfate transport, we compared sulfate transport of WT *SLC26A1* with the variants p.Pro237Leu, p.Leu348Pro, p.Thr185Met, and p.Ser358Leu in the oocyte expression system. The 4 variants were selected based on Supplemental Table 2: variant Pro237Leu had shown the largest effect size on plasma sulfate levels; Leu348Pro is the only *SLC26A1* variant for which a disturbance of human sulfate homeostasis has previously been reported in a population study, but without experimental validation (24); and Thr185Met and Ser358Leu, which were reported in the compound heterozygous state in 1 patient with kidney stones in a previous publication (14) but were detected individually in carriers of the GCKD study.

**Figure 8:**
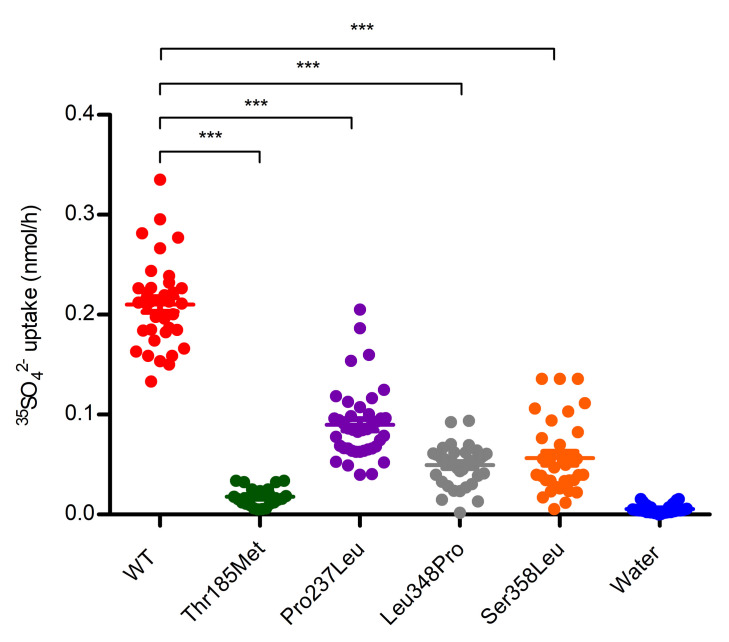
Functional evaluation of *SLC26A1* variants identified in the GCKD study. SLC26A1-mediated SO_4_^2−^ uptake by oocytes previously water injected or injected with 10 ng of cRNA encoding WT SLC26A1 or the indicated mutants (Thr185Met, Pro237Leu, Leu348Pro, and Ser358Leu). Uptakes were carried out from a bath solution containing 1 mM SO_4_^2−^ for 15 minutes. The experiments were performed with oocytes from 4 different frogs (and 3 different cRNA preparations) with a total number of 36 WT, 23 Thr185Met, 41 Pro237Leu, 41 Leu348Pro, 33 Ser358Leu, and 40 water injected. Data are presented as mean ± SEM. ***P < 0.001 by 1-way ANOVA with Bonferroni’s multiple-comparison test.

As shown in Figure 8, a substantially reduced sulfate transport was detected for all 4 variants, with the greatest reduction in the case of Thr185Met and lowest for Pro237Leu. When coexpressed 1:1 with WT SLC26A1 at constant total amount of cRNA per oocyte, the mutant with the most prominent effect, Thr185Met, reduced sulfate transport down to approximately 25% (Supplemental Figure 4). Since transporters of the SLC26 gene family may assemble into functional dimers (25), which predicts the presence of 25% WT/WT, 50% WT/mutant, and 25% mutant/mutant transporters upon 1:1 coexpression of WT with the mutant, the 75% reduction in transport is consistent with a strong dominant negative effect of the p.Thr185Met mutant.

## Discussion

Our patient exhibited what we believe is a novel homozygous *SLC26A1* mutation (Leu275Pro) resulting in urinary sulfate wasting and low plasma sulfate, which is in line with the hyposulfatemia and elevated fractional excretion of sulfate observed in *Slc26a1*-null mice (10). Functional expression studies established that the mutant human transporter is encoded by a loss-of-function allele resulting in a substantial defect in sulfate transport ability. The importance of *SLC26A1* in sulfate transport was further corroborated by significantly lower plasma sulfate levels among heterozygous carriers of rare, deleterious *SLC26A1* variants detected through whole-exome sequencing in a large observational study, which confirms and expands a prior report that a genotyped *SLC26A1* missense variant (Leu348Pro) was related to sulfate homeostasis (24). Functional relevance of the alleles identified in the population study, including the aforementioned Leu348Pro, was experimentally validated in expression studies.

Recently, rare loss-of-function variants of *SLC13A1* have been demonstrated to be associated with hyposulfatemia, back pain, and intervertebral disc disorder, another example of painful cartilage disease (7). It is therefore very likely that the hyposulfatemia was responsible for an otherwise unexplained perichondritis of a mechanically highly stressed joint in our patient. Of note, the symptoms had started at the end of the patient’s first pregnancy, a condition with increased sulfate demand (18, 26). As demonstrated in functional analyses in oocytes transfected with mutant *SLC26A1*, transport activity was not completely abrogated by the patient mutation, which may explain why our patient did not manifest more severe abnormalities of her musculoskeletal system, except a slightly reduced bone-mineral density in dual-energy X-ray absorptiometry, consistent with a previous report of individuals with lower sulfate levels (24).

There are few data about *SLC26A1* and sulfate-related disorders in humans (24). In *Slc26a1*-knockout mice, disturbances of sulfate homeostasis were consistently described (10, 11), but musculoskeletal abnormalities were not reported (4, 10, 11, 13). However, they were also not explicitly examined and might easily have been overlooked.

The GCKD cohort was not designed to assess potential hyposulfatemia-related musculoskeletal disorders, including joint or back pain, arthrosis, or fibromyalgia. Therefore, our study only allows correlation of carrier status with plasma sulfate levels. Moreover, hyposulfatemia-related symptoms might be very subtle as early arthrosis or joint pain, and might be easily overlooked in clinical practice if they are not actively screened for, which underlines the need for subsequent clinical studies to determine the relationship between *SLC26A1* and musculoskeletal health with more certainty.

Gee and colleagues detected biallelic mutations in *SLC26A1* in 2 unrelated individuals with calcium oxalate kidney stones (14), in whom urine oxalate was normal to mildly elevated. In our patient, an analysis of the isolated kidney stone was not available, but repeated measurements of oxalate in the urine did not show reproducible hyperoxaluria. In such a setting, the increased fractional excretion of sulfate might also serve as a driver of kidney stone formation (27). Unfortunately, in the 2 patients with nephrolithiasis and *SLC26A1* mutations described by Gee et al., plasma and urinary sulfate levels were not measured. A recent study in *Slc26a1^–/–^* mice confirmed hyposulfatemia and renal sulfate wasting but failed to demonstrate hyperoxaluria and hyperoxalemia (13). Thus, the role of SLC26A1 may be predominantly related to sulfate homeostasis, whereas its role in oxalate homeostasis requires further study. Our findings are supported by kinetic studies that concluded that SLC26A1 would not mediate substantial influx of oxalate under physiological conditions (28).

In the GCKD study, heterozygous carriers of potentially damaging *SLC26A1* variants did not have a higher prevalence of self-reported kidney stones compared to noncarriers (*P* > 0.1). This result does not contradict the data from Gee and colleagues (14), as all variant carriers in the GCKD cohort were heterozygous, whereas the individuals reported by Gee et al. were biallelic, as was our patient with a *SLC26A1* mutation. Thus, the data presented allow *SLC26A1* to remain a potential risk factor for nephrolithiasis. However, the underlying pathophysiological mechanism(s), including the relative contribution of urinary oxalate and sulfate to stone formation, may deserve reevaluation.

Strengths of our study include support for the role of SLC26A1 as a physiologically important human sulfate transporter from 2 independent, complementary lines of genetic evidence; while unexplained musculoskeletal symptoms in a single patient led us to the identification of a homozygous loss-of-function mutation in the gene that was confirmed through functional studies, the physiological role of SLC26A1 in sulfate homeostasis was supported by the aggregate effect of rare, heterozygous coding variants in *SLC26A1* that collectively are abundant in the population. The latter finding was substantially bolstered by our functional analysis, which revealed marked impairment of sulfate transport for several of these variants. The dominant negative effect of the p.Thr185Met mutation is proof of principle that even heterozygous carriers of a damaging variant may present with clearly reduced sulfate transport that in turn leads to reduced plasma sulfate concentrations.

We would like to emphasize that the data presented do not meet the criteria for ClinGen gene-phenotype attribution. At present, there is not sufficient evidence to define a Mendelian disorder. The correlation between biallelic deleterious variants of *SLC26A1* and potential clinical features (urolithiasis and/or cartilage or bone disorders) deserves further clarification. However, our work paves the way for future studies to establish whether the frequency of the various *SLC26A1* variants identified in this work are more common in individuals with cartilage and bone disease as compared with the general population.

Our findings showcase that the significance of a presumably new genetic finding might be derived from a single case if the gene’s impact on the phenotype can be matched through orthogonal evidence from population-based studies, rather than necessitating the identification of a second case or family. As molecular genome-wide screening, including next-generation sequencing, is being performed more frequently (15), this approach might be a helpful tool for future clinical practice.

In conclusion, the present work demonstrates that *SLC26A1* is a major determinant of sulfate homeostasis in humans. In view of recent evidence linking sulfate homeostasis to back pain and intervertebral disc disorder (7), our study identifies SLC26A1 as a potential target for modulation of musculoskeletal health.

## Methods

### Genetic information

#### Patient testing.

After written informed consent from the patient and her family, also regarding publication, we performed whole-exome sequencing and trio analysis (index patient and parents) with Sanger sequencing according to the requirements of the German Gene Diagnostic Act (GenDG).

#### Population study.

The GCKD study enrolled 5,217 participants from 2010 to 2012, as described previously (22). In brief, participants were eligible if their eGFR was 30–60 mL/min/1.73 m^2^ at screening, or if they had relevant proteinuria (urinary albumin to creatinine ratio >300 mg/g or proteinuria equivalent) in the presence of higher eGFR. The study was registered in the national registry for clinical studies (DRKS 00003971) and approved by the relevant ethics committees.

### Genetic testing

### Patient

### Whole-exome sequencing and trio analysis (index patient and parents)

Genomic DNA was extracted from peripheral blood using the Chemagic Star DNA Blood 400 Kit from PerkinElmer. Whole-exome sequencing was performed (SYNLAB MVZ Humane Genetik Munich) for the patient and her parents. Paired-end 150-bp whole-exome sequencing was performed on the NextSeq 500 platform (Illumina) using the Human Core Exome kit (TWIST Bioscience). NGS raw data processing, genomics data management, and variant interpretation including single-nucleotide variants (SNVs) and copy number variants (CNVs) was performed using the varvis genomics platform (Limbus Medical Technologies). Generation of NGS data via trio analyses was done assuming an autosomal recessive mode of inheritance and potentially pathogenic de novo changes. Only variants were considered that at the time of analysis had a minor allele frequency of less than 0.1% and in addition had a probability for a causal association with the clinical phenotype of the patient. Evaluation of the pathogenicity of identified SNVs and CNVs was carried out according to the American College of Medical Genetics guidelines (29). Public databases (Decipher, ClinVar, ClinGen, LOVD) and different in silico prediction programs (SIFT, PolyPhen2, Align GVGD, Mutation Taster, SpliceSiteFinder-like, MaxEntScan, NNSPLICE, GeneSplicer) were used for the interpretation of the identified variations. The *SLC26A1* variant identified in the patient has been deposited in ClinVar under the submission ID SUB12301997. Next-generation sequencing data can be provided on individual request.

### Sanger sequencing (brother of index patient)

The *SLC26A1* sequence region in which the identified pathogenic variant is localized was PCR amplified under standard conditions with Thermo-Start DNA Polymerase (Thermo Fisher Scientific) and the primers SLC26A1-001: CTGACCTCGCAGCTCAAAC (forward primer) and SLC26A1-002: CACCACGATGACCAGCAG (reverse primer). Subsequently, Sanger sequencing was performed (*SLC26A1* reference sequence NM_022042.4). Sequence reactions were performed with Big Dye Terminator Chemistry v1.1 (Applied Biosystems) on the Applied Biosystems 3730xl DNA Analyzer. Sequences were analyzed with the Sequence Pilot algorithm v5.1.0 (JSI Medical Systems).

### Study population (GCKD study)

Genomic DNA from blood samples collected at the enrollment visit underwent paired-end 100-bp whole-exome sequencing at Human Longevity Inc., using the Illumina NovaSeq 6000 platform and IDT xGen v1 capture kit. On average, greater than 97% of consensus coding sequence (CCDS) (30) release 22 had at least 10× coverage, and average coverage of the CCDS achieved 141-fold read depth.

The exome sequences were processed at AstraZeneca from their unaligned FASTQ state in a custom-built cloud compute platform running the Illumina DRAGEN Bio-IT Platform, and germline Pipeline v3.0.7 was adopted to align reads to the GRCh38 reference genome and perform variant calling (23).

Variants were annotated using the variant effect predictor (VEP) v101 (31). Using the standard settings, we annotated the canonical transcript, gene symbols, and included frequencies from the Genome Aggregation Database (gnomAD v2.1; https://gnomad.broadinstitute.org/). We used VEP plugins to add the REVEL score (v2020-5) (32) and the CADD score (v3.0) (33). Loss-of-function variants were downgraded using the LoFtee VEP plugin (v2020-8) (34). Multiple in silico scores were added using information from the dbNSFP (v4.1a) (35).

During sample-level quality control, samples with an estimated VerifyBamID freemix (36) contamination level of greater than 4%, samples of non-European ancestry, duplicate samples, and samples with mismatch of reported and genetically predicted sex were excluded.

When the GCKD study was initiated in 2009, the recruitment was restricted to individuals of European ancestry (22). The rationale at the time was that the proportion of individuals of non-European ancestry in the recruiting practices was rather low, and the investigators were concerned about potential biases in GFR estimation because the employed estimating equations contained a race term. Using genome-wide genetic data, we computed genetic ancestry and excluded a small number of individuals because they showed outlying values along any of the first 10 principal components from a principal component analysis in order to maximize sample homogeneity, as described previously (37). Furthermore, we only kept samples with available high-quality DNA microarray genotype data, resulting in 4,868 samples with available whole-exome sequencing data.

### Laboratory parameters

The measurement of laboratory parameters was performed following standard routine, with exceptions as listed below.

### Measurement of plasma oxalate concentration

Plasma oxalate concentration was measured enzymatically using an oxalate oxidase assay as previously described (Trinity Biotech) (38).

### Measurement of plasma and urinary sulfate concentrations

#### Patient.

Venous blood samples were collected into Vacuette heparinized plasma tubes (Greiner Bio-One GmbH), immediately put on wet ice, and centrifuged for 10 minutes at 1,800*g*. The supernatant was aliquoted and stored at –80°C. For measurement of urinary sulfate, 5 mL of urine was collected at the same time the blood was collected, put on wet ice, and then stored at –80°C. After thawing, free sulfate levels in plasma and urine were measured using an AB Sciex 4000 Q-TRAP or 5000 LC-MS/MS system operating in negative ionization mode. Samples were introduced via flow-injection analysis, and the transitions *m*/*z* 97→80 and *m*/*z* 99→82 were monitored for sulfate (SO_4_) and ^34^SO_4_ (serving as internal standard, mass shift is +2), respectively. Urinary samples were prepared by an initial 100-fold dilution of the original urine with distilled water. Twenty microliters of the diluted urine was mixed with 2 nmol of ^34^SO_4_, and the obtained mixture was purified through a 10 kDa filter (Amicon) by centrifugation. From the final filtrate, 10 μL was introduced to the mass spectrometer by flow injection analysis. Plasma samples were prepared by mixing 20 μL of plasma with 2 nmol of ^34^SO_4_ and 180 μL of purified water, followed by a deproteinization step using a 10 kDa filter (Amicon) by centrifugation. From the final filtrate, 1 μL was introduced to the mass spectrometer by flow injection analysis (10 mM formic acid adjusted to pH 8.75 with ammonia). Aqueous calibrators with known amounts of sulfate were used for quantification. The obtained peak-area ratios of the trace of sulfate related to the trace of the internal standard were then used for the estimation of the free sulfate levels in the examined body fluids.

#### Population.

Sulfate was measured in plasma collected at the GCKD study enrollment visit as part of a nontargeted MS-based metabolomics panel (Metabolon HD4) at Metabolon described in detail previously (39). Sulfate levels were quantified as part of the LC/MS Neg platform, with an intra-assay coefficient of variation of 6.9%, and available for 5,144 participants.

### Measurement of oxalate and sulfate transport in Xenopus laevis oocytes

### Molecular biology

Human *SLC26A1* (NM_022042) subcloned into the *Xenopus* oocyte expression vector pXT7 (pXT7-SLC26A1) was provided by Seth Alper (Harvard Medical School, Boston, Massachusetts, USA). The various point mutations were introduced by QuikChange II (Agilent) site-directed mutagenesis kit using specific mutagenic oligonucleotides (for Leu275Pro, forward 5′-GGCGGTGCTGCCAGCCGCGAAGG-3′ and reverse 5′-CCTTCGCGGCTGGCAGCACCGCC-3′; for Thr185Met, forward 5′-CGCCACCGCCCTCATGCTGATGACC-3′ and reverse 5′-GTCATCAGCATGAGGGCGGTGGCG-3; for Pro237Leu, forward 5′-CGTGCGGATCCTGCGGCACCAGG-3′ and reverse 5′-CCTGGTGCCGCAGGATCCGCACG-3′; for Leu348Pro, forward 5′-TGCCGTGGCCCCGGCCCTCGTGG-3′ and reverse 5′-CCACGAGGGCCGGGGCCACGGCA-3′; and for Ser358Leu, forward 5′-TGCCGCCTTCTCCATCTTGCTGGCGGA-3′ and reverse 5′-TCCGCCAGCAAGATGGAGAAGGCGGCA-3′. An HA epitope was inserted by PCR mutagenesis into the second extracellular loop, after Pro^155^ of WT SLC26A1 and the Leu275Pro mutant. All constructs were confirmed by sequencing the complete open reading frame.

### Expression of cRNA in Xenopus oocytes

Oocytes were injected with cRNA (10 ng) transcribed with the mMessage Machine T7 kit (Thermo Fisher Scientific) according to the manufacturer’s instructions after linearization of the plasmid with XbaI. Oocytes injected with 10 ng of cRNA or water (as a control) were maintained at 17.5°C in ND96 buffer containing penicillin/streptomycin (100 μg/mL) for 2 days before use.

### Isotopic influx

Oocytes were incubated for 15 minutes at room temperature in ND96 bath solution (in mM): 96 NaCl, 2 KCl, 1.8 CaCl_2_, 1 MgCl_2_, and 5 HEPES, pH 7.40, containing 1 mM (13 μCi/mL) ^35^SO_4_^2−^ (Biotrend) or 1 mM (2.5 μCi/mL) ^14^C-oxalate (ViTrax). For ^14^C-oxalate influx experiments, ND96 bath solution was nominally Ca^2+^ and Mg^2+^ free. Influx experiments were terminated with 4 washes in ice-cold ND96, followed by oocyte lysis in 150 μL of 2% sodium dodecyl sulfate (SDS). Control experiments indicated that at 15 minutes uptake was still in a near-linear range. Duplicate 10 μL aliquots of influx solution (in 150 μL 2% SDS) were used to calculate specific activities of radiolabeled substrate. Single oocyte uptake was calculated from oocyte-associated counts per minute (cpm) and bath specific activity. All samples were analyzed by scintillation counting with 2 mL of the scintillation cocktail Aquasafe 300 plus (Zinsser Analytics) in a Tri-Carb 2810 TR β-scintillation counter (PerkinElmer).

### Western blotting

Oocytes were homogenized in a buffer containing (in mM): 20 Tris-HCl pH 7.4, 140 NaCl, 2 EDTA, and protease inhibitors (4 mM Pefabloc, Complete EDTA-free protease inhibitor mixture, Roche). The homogenate was cleared by centrifugation for 10 minutes at 1,000*g* twice, and membrane fractions were pelleted from the cleared homogenate by ultracentrifugation for 30 minutes at 100,000*g*. The membrane pellet was resuspended by sonication in 50 mM Tris-HCl, pH 6.8, 140 mM NaCl, 0.5 mM EDTA, 1% SDS (w/v), and 1% Triton X-100 (w/v) with protease inhibitors. Equal amounts of protein (30 μg) were separated via SDS-PAGE and blotted onto a nitrocellulose membrane. Western blots were probed with monoclonal rabbit anti-HA tag antibody (Cell Signaling Technology, catalog 3724) or rabbit anti-SLC26A1 (Novusbio, catalog NBP1-5908). ImageJ (NIH) was used for quantification of the Western blots. Protein levels were normalized to actin (rabbit polyclonal; Sigma-Aldrich, catalog A3066) on the same blot.

### Immunofluorescence

Two days after injection with cRNA (10 ng) encoding WT or mutant HA-tagged SLC26A1, oocytes were fixed with 1% paraformaldehyde (PFA) in phosphate-buffered saline (PBS) for 10 minutes at 4°C and washed 3 times with cold PBS. Oocytes were blocked for 1 hour at room temperature in PBS containing 1% bovine serum albumin (PBS-BSA) and subsequently incubated for 1 hour at room temperature with anti-HA tag antibody (Cell Signaling Technology, catalog 3724) in PBS-BSA, after which the oocytes were washed 3 times with cold PBS. Antibody-labeled oocytes were then incubated for 1 hour at room temperature with secondary antibodies coupled to Alexa Fluor 555 (Molecular Probes), washed 3 times with cold PBS, and stored at 4°C until imaging. Confocal images were taken with a Zeiss LSM880 microscope using ZEN software. For the quantification of immunofluorescence microscopy images, ImageJ was used.

### Statistics

To investigate the combined effects of rare exonic genetic variants in *SLC26A1* on plasma sulfate levels in the GCKD study, we performed an aggregate variant test using data from 4,708 GCKD study participants with all available data. The qualifying variants aggregated in *SLC26A1* were selected using annotations from VEP v101 (31). All variants with minor allele frequency of less than 0.5% that were predicted to be either high-confidence loss-of-function variants or missense variants with a MetaSVM score (40) greater than 0 or inframe nonsynonymous variants with a fathmm-XF-coding score (41) greater than 0.5 were included for gene-based testing (*N* = 43). Aggregate variant testing was performed with the burden test as implemented in the SeqMeta R package version 1.6.7 (42). Sulfate levels in plasma were rank-based, inverse normal transformed prior to analysis, and adjusted for age, sex, ln(eGFR), serum albumin, and the first 3 genetic principal components. The statistical significance threshold for the burden test was set to a *P* value of less than 0.05, corresponding to the test of 1 candidate gene.

Concerning the experiments with *Xenopus laevis* oocytes, the statistical analyses are always indicated in the figure legends (1-way ANOVA with Bonferroni’s multiple-comparison test or 2-tailed, unpaired *t* test, where appropriate). A *P* value of less than 0.05 was considered significant.

### Study approval

The GCKD study was conducted according to Declaration of Helsinki principles and was approved by all appropriate institutional review board and ethics committees. All participants provided written informed consent prior to inclusion in the study.

## Author contributions

FK, AP, and AK developed the research and design of the study. Resources, methodology, and supervision were provided by FK, PSA, TJJ, AK, FCL, and KUE. AP, FK, DGF, and KZ were involved in different aspects of clinical care for the patient, including interpretation of imaging. KILC performed and analyzed the functional experiments. AW and DECS performed extended patient-related laboratory measurements and analyses. Biostatical analysis and interpretation were done by NS, MW, and AK. Data were interpreted by FK, AP, PSA, TJJ, KILC, AK, and JMVDK. AP, KILC, NS, AK, and KZ created the figures. AP, PSA, AK, and FK contributed to the writing of the first manuscript draft, which was approved and edited by all co-authors. FK, AK, and TJJ are co–senior authors. AP and KILC are co–first authors. Authorship order among first authors was decided based on AP initiating the project.

## Supplementary Material

Supplemental data

## Figures and Tables

**Table 1:**
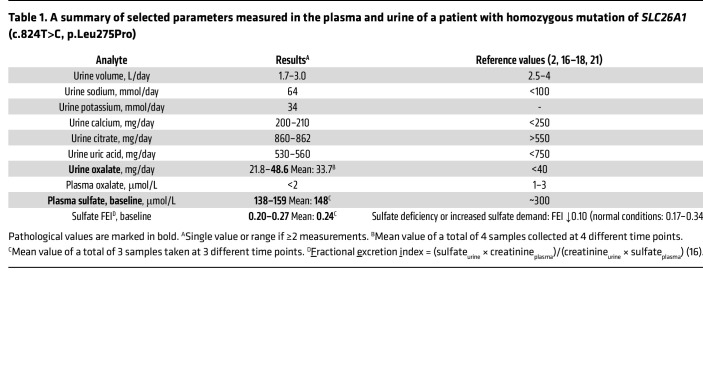
A summary of selected parameters measured in the plasma and urine of a patient with homozygous mutation of *SLC26A1* (c.824T>C, p.Leu275Pro)

## Version 1 02/01/2023

Electronic publication
