# Supplementary material for: SLC26A1 is a major determinant of sulfate homeostasis in humans
Source: J Clin Invest. 2023 Feb 1;133(3):e161849. doi: 10.1172/JCI161849 (PMC9888379; doi:10.1172/JCI161849)
Supplement: Supplemental data [file jci-133-161849-s152.pdf]

Supplemental Material

SLC26A1 is a major determinant of sulfate homeostasis in humans

Table of Contents

Supplementary Table S1. Complete results of plasma and urinary sulfate concentrations in a patient with homozygous mutation of *SLC26A1* (p.Leu275Pro) compared to results in one heterozygous carrier of p.Leu275Pro (mother), and a group of unrelated controls.

Supplementary Table S2. List and annotation of qualifying variants in *SLC26A1* included in aggregate variant testing with plasma sulfate levels in the GCKD study.

Supplementary Figure S1. Effect of epitope insertion on SLC26A1.

Supplementary Figure S2. P-values from aggregate tests based on sequential addition or omission of *SLC26A1* variants with plasma sulfate levels in the GCKD study.

Supplementary Figure S3. Distribution of plasma sulfate levels in heterozygous carriers of potentially damaging *SLC26A1* variants with and without reduced eGFR in the GCKD study.

Supplementary Figures S4. Dominant negative effect of mutant SLC26A1 (Thr185Met).

Supplementary References.

Supplementary Table S1. Complete results of plasma and urinary sulfate concentrations in a patient with homozygous mutation of *SLC26A1* (p.Leu275Pro) compared to results in one heterozygous carrier of p.Leu275Pro (mother), and a group of unrelated controls.[a]

	**Patient**	**Heterozygous control**	**Controls**[a]	**% of controls**
Mean plasma sulfate [µmol/L]	148[b]	340 (n=1)	381	0.39
Plasma sulfate range [µmol/L]	138-159	-	303-483	
Mean urinary sulfate [µmol/L]	8820	16007	N/A	N/A
Urinary sulfate range [µmol/L]	4201-17286	-	N/A	
Mean FEI[c]	0.24[b]	0.22	N/A	N/A
FEI range	0.20-0.27	-	N/A	

N/A=not available

[a] Plasma control samples are from 10 anonymous individuals aged 2-18 years. The samples are residual material taken for routine clinical chemistry analysis.

[b] Mean value of a total of 3 samples taken at 3 different timepoints.

[c] FEI (fractional excretion index)=[(sulfate (urine) x creatinine (plasma)]/[creatinine (urine) x sulfate (plasma)]. (1)

Supplementary Table S2. List and annotation of qualifying variants in *SLC26A1* included in aggregate variant testing with plasma sulfate levels in the GCKD study.

The listed effect sizes, standard errors and P-values summarize each variant's association with plasma sulfate levels, with the effect direction corresponding to the effect of the minor alternative allele. The annotation is based on transcript ENST00000361661 (canonical transcript) and obtained from VEP (Methods). Aggregate variant testing that combines the effects of all qualifying variants in *SLC26A1* on plasma sulfate levels using a burden test yielded a significant P-value of 3.01e-05 and an effect size of -0.26 with a standard error of 0.06 (SE: standard error; MAC: minor allele count.)

Variant name	SNP ID if known	Position (hg38)	Reference allele	Alternative allele	Alt. allele frequency	Effect size	SE	P-value	MAC	Variant consequence	Protein position	Amino acids
4:989033:C:A	rs146466185	989033	C	A	0,0034	-0,02	0,13	8,74E-01	32	missense	636	D/Y
4:989072:C:T	rs747328202	989072	C	T	0,0001	0,23	0,73	7,47E-01	1	missense	623	D/N
4:989254:G:A	rs201503661	989254	G	A	0,0002	-0,52	0,51	3,08E-01	2	missense	562	T/M
4:989317:C:T	rs376289512	989317	C	T	0,0002	-0,33	0,51	5,24E-01	2	missense	541	R/H
4:989396:G:A	rs375537240	989396	G	A	0,0003	-1,01	0,42	1,57E-02	3	missense	515	R/C
4:989402:G:T	rs775080599	989402	G	T	0,0001	0,30	0,73	6,75E-01	1	missense	513	L/M
4:989420:G:A	rs368806302	989420	G	A	0,0001	0,21	0,73	7,74E-01	1	missense	507	R/C
4:989428:C:T	rs201106151	989428	C	T	0,0001	-0,61	0,73	4,00E-01	1	missense	504	R/H
4:989491:A:T	rs387907487	989491	A	T	0,0001	-0,87	0,73	2,32E-01	1	missense	483	L/Q
4:989527:G:A	rs377075470	989527	G	A	0,0001	0,87	0,73	2,35E-01	1	missense	471	A/V
4:989536:G:A	rs923099050	989536	G	A	0,0001	-0,65	0,73	3,71E-01	1	missense	468	P/L

4:989557:G:A	rs376705237	989557	G	A	0,0001	-1,25	0,73	8,49E-02	1	missense	461	P/L
4:989575:C:A	rs371025483	989575	C	A	0,0001	-0,46	0,73	5,28E-01	1	missense	455	R/L
4:989596:A:G		989596	A	G	0,0001	-0,70	0,73	3,32E-01	1	missense	448	V/A
4:989623:C:A	rs148721282	989623	C	A	0,0001	0,45	0,73	5,39E-01	1	missense	439	R/L
4:989695:G:A	rs200470975	989695	G	A	0,0009	-0,49	0,26	5,86E-02	8	missense	415	S/F
4:989708:G:A	rs554753768	989708	G	A	0,0002	-0,28	0,51	5,85E-01	2	missense	411	R/W
4:989714:C:T	rs747534272	989714	C	T	0,0002	0,11	0,51	8,29E-01	2	missense	409	G/S
4:989750:C:T	rs572968482	989750	C	T	0,0002	0,21	0,51	6,88E-01	2	missense	397	A/T
4:989786:C:T	rs146487540	989786	C	T	0,0001	0,20	0,73	7,85E-01	1	missense	385	V/M
4:989866:G:A	rs148832260	989866	G	A	0,0004	-0,46	0,36	2,09E-01	4	missense	358	S/L
4:989879:C:T	rs756246013	989879	C	T	0,0001	0,76	0,73	2,96E-01	1	missense	354	A/T
4:989896:A:G	rs148386572	989896	A	G	0,0026	-0,53	0,15	3,00E-04	24	missense	348	L/P
4:989965:G:A	rs202242030	989965	G	A	0,0001	-0,82	0,73	2,57E-01	1	missense	325	T/M
4:989999:G:A	rs750594086	989999	G	A	0,0001	-1,31	0,73	7,05E-02	1	missense	314	R/C
4:990080:G:A	rs201572215	990080	G	A	0,0005	-0,12	0,32	7,08E-01	5	missense	287	R/C
4:990091:C:T	rs776919627	990091	C	T	0,0002	-0,50	0,51	3,26E-01	2	missense	283	R/H
4:990155:A:G	rs1219843112	990155	A	G	0,0001	-0,93	0,73	1,99E-01	1	missense	262	C/R
4:990179:G:A	rs370834972	990179	G	A	0,0002	-0,28	0,52	5,93E-01	2	missense	254	R/C
4:990229:G:A	rs778482338	990229	G	A	0,0001	-1,59	0,73	2,86E-02	1	missense	237	P/L
4:990239:C:T	rs367665258	990239	C	T	0,0001	-0,95	0,73	1,90E-01	1	missense	234	V/M
4:991150:G:A	rs139024319	991150	G	A	0,0002	-1,11	0,51	3,08E-02	2	missense	185	T/M

4:991169:G:A	rs370208644	991169	G	A	0,0002	-0,18	0,51	7,28E-01	2	missense	179	R/C
4:991270:C:A	rs764183197	991270	C	A	0,0001	-0,14	0,73	8,45E-01	1	missense	145	G/V
4:991288:C:T	rs148314592	991288	C	T	0,0001	0,39	0,73	5,90E-01	1	missense	139	R/Q
4:991289:G:A	rs552862956	991289	G	A	0,0001	0,57	0,73	4,33E-01	1	missense	139	R/W
4:991348:C:T	rs368990025	991348	C	T	0,0001	-0,26	0,73	7,22E-01	1	missense	119	R/Q
4:991356:GC:G	rs756881822	991356	GC	G	0,0002	-0,66	0,51	1,98E-01	2	frame-shift	116	G/X
4:991369:T:C	rs769261621	991369	T	C	0,0001	-0,15	0,73	8,42E-01	1	missense	112	Y/C
4:991450:G:A	rs139918804	991450	G	A	0,0001	-0,65	0,73	3,75E-01	1	missense	85	P/L
4:991466:C:T	rs374288131	991466	C	T	0,0002	0,56	0,51	2,75E-01	2	missense	80	G/S
4:991522:C:T	rs138449255	991522	C	T	0,0007	0,06	0,27	8,26E-01	7	missense	61	R/H
4:991538:C:T	rs142573758	991538	C	T	0,0003	0,27	0,42	5,26E-01	3	missense	56	A/T

Supplementary Figure S1: Effect of epitope insertion on SLC26A1.

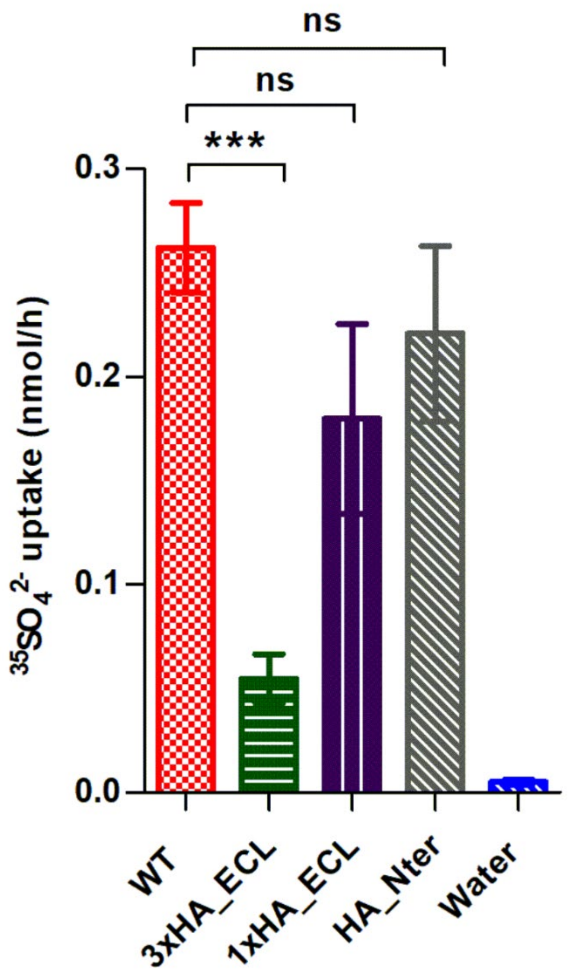

HA-epitopes were inserted into the second extracellular loop (ECL) of SLC26A1 to determine surface expression in non-permeabilized cells (see Figure 5). Control experiments to determine effect of epitope addition on $^{35}SO_4^{2-}$ transport across the plasma membrane. 3 HA epitopes were inserted after proline 148 (construct 3xHA_ECL), or 1 HA epitope after proline 155 (1xHA_ECL). A previously described (2) mutant carrying an HA epitope at the cytoplasmic N-terminus served as control (HA_Nter). The 1xHA_ECL construct, which lacked significant effect on $^{35}SO_4^{2-}$ fluxes, was chosen for further experiments in Figure 5. The SO_4^{2-} uptake experiments were performed with oocytes from two different frogs (and 2 different cRNA preparations) with a total number of 23 (WT), 15 (3xHA_ECL), 18 (1xHA_ECL), 18 (HA_Nter), and

23 water-injected oocytes. Error bars, means ± SEM, One-way ANOVA, Bonferroni multiple comparison test ***$p < 0.001$.

Supplementary Figure S2. P-values from aggregate tests based on sequential addition or omission of *SLC26A1* variants with plasma sulfate levels in the GCKD study.

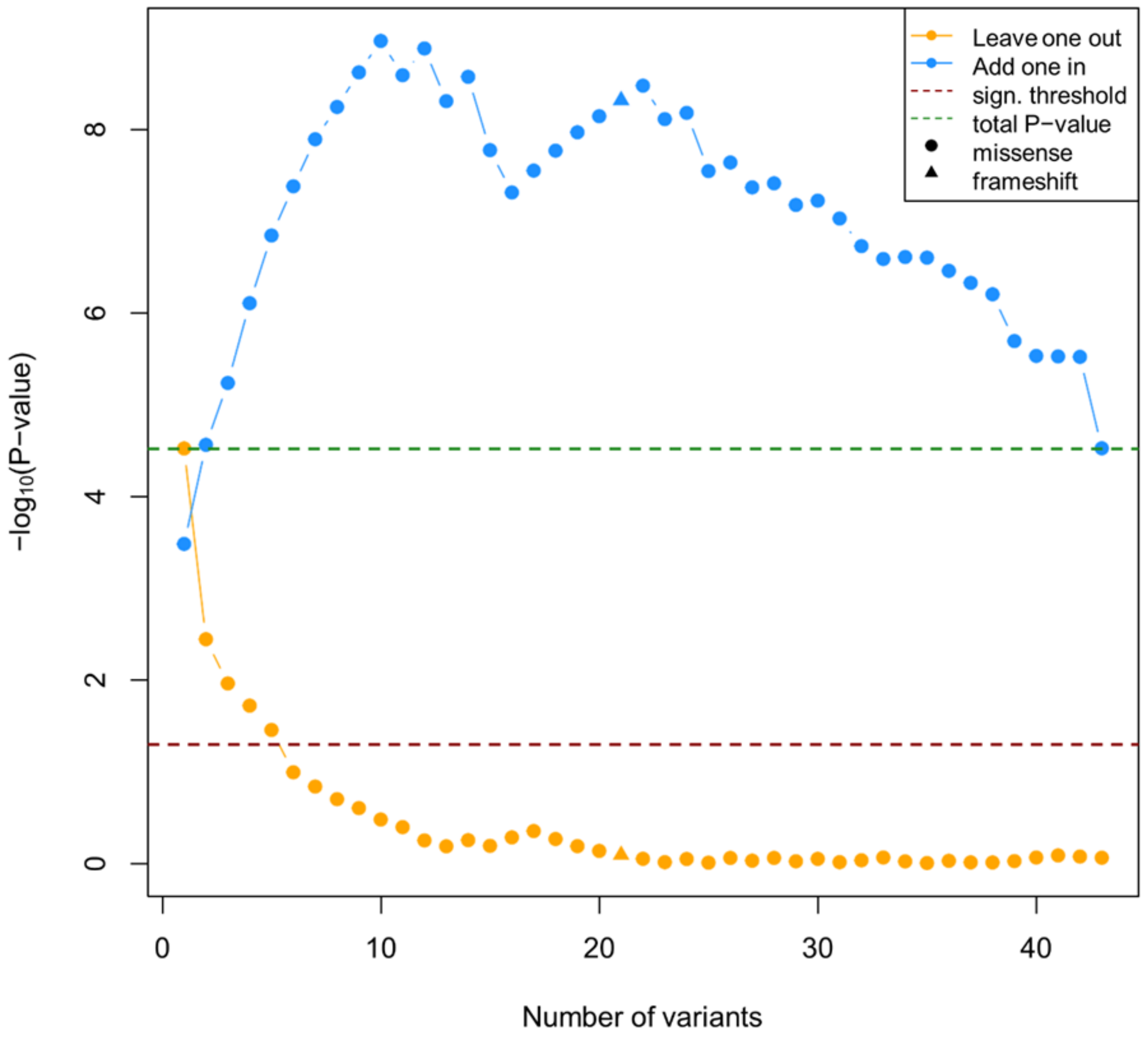

The blue points describe the development of the -$\log_{10}$(P-value) (y-axis) of aggregate variant tests, where P-value ranked variants are added consecutively to the set of variants used for aggregate testing, starting from the variant with the lowest individual P-value. The number of variants included in the test is given on the x-axis. Hence the leftmost blue point represents the -$\log_{10}$(P-value) of the test where only the variant with the lowest individual P-value is included. The orange points describe the development

of the -$\log_{10}$(P-value) of aggregate variant tests, where the variants sorted by P-value are omitted successively, starting with the variant with the lowest individual P-value. Hence the leftmost orange point represents the -$\log_{10}$(P-value) of all qualifying variants. The shape of the variants represents their predicted consequence. The green dashed line denotes the -$\log_{10}$(3.01e-05) of the aggregate variant test including all 43 qualifying variants in *SLC26A1*. The red dashed line marks the significance threshold -$\log_{10}$(0.05).

Supplementary Figure S3. Distribution of plasma sulfate levels in heterozygous carriers of potentially damaging *SLC26A1* variants with and without reduced eGFR in the GCKD study.

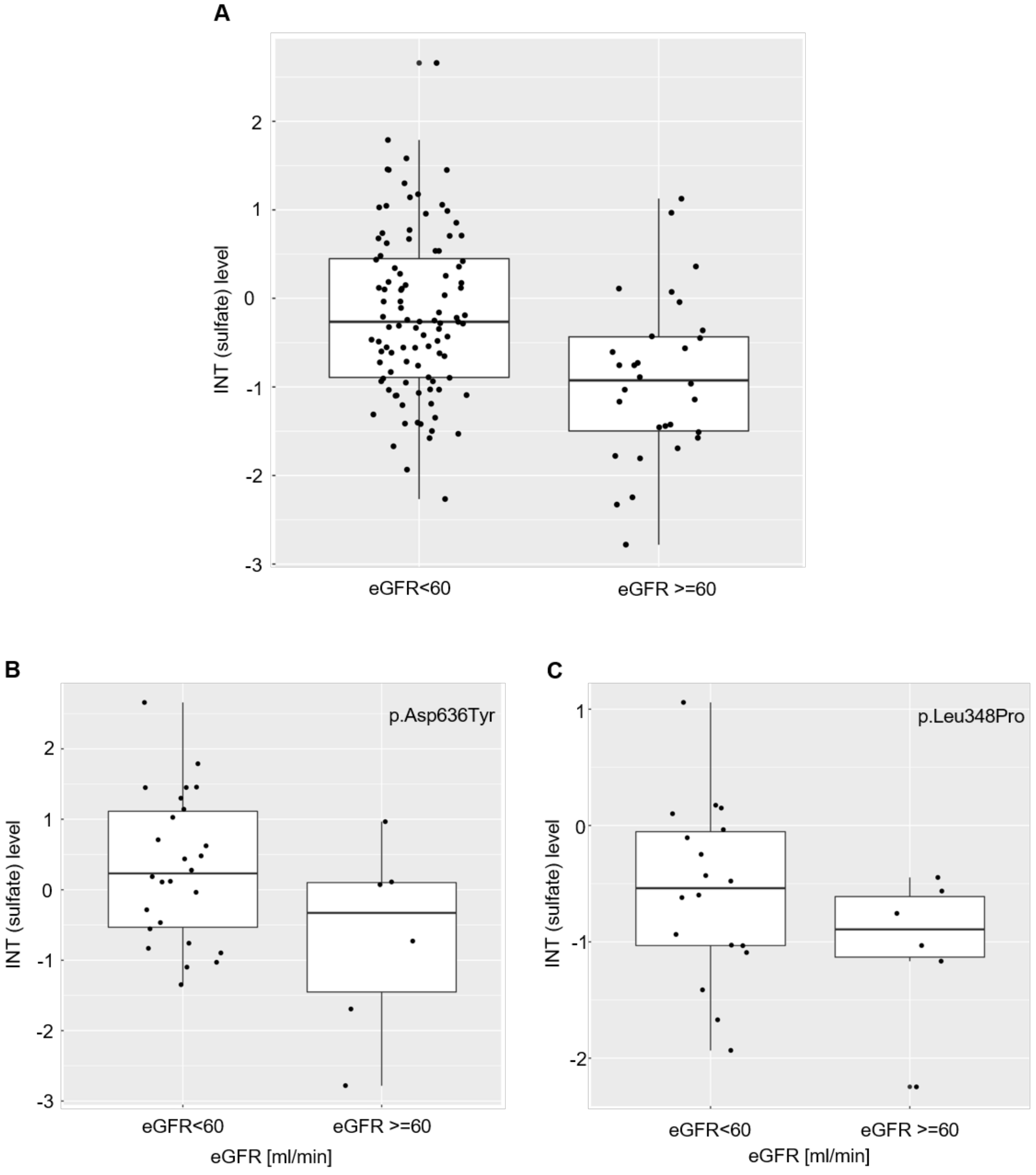

Within the group of 130 heterozygous carriers of 43 potentially damaging *SLC26A1* variants in the GCKD study, individuals with an eGFR ≥ 60 ml/min/1.73m^2 (x-axis) had significantly lower average plasma sulfate levels (y-axis) than those with an eGFR <

60 ml/min/1.73m^2 (unpaired t-test, two-tailed, $p<0.001$, n=130) (**A**). This effect was reproducible when focusing only on persons carrying the same variant, namely (**B**) p.Asp636Tyr (p=0.057, n=32), and (**C**) p.Leu348Pro (p=0.18, n=24). Unpaired T-test, two-tailed. Sulfate levels on the y-axis are presented after inverse normal transformation (INT). The boxes range from the 25th to the 75th percentile of sulfate levels, the median is indicated by a line, and whiskers end at last observed value within 1.5*(interquartile range) away from box.

Supplementary Figure S4. Dominant negative effect of mutant SLC26A1 (Thr185Met).

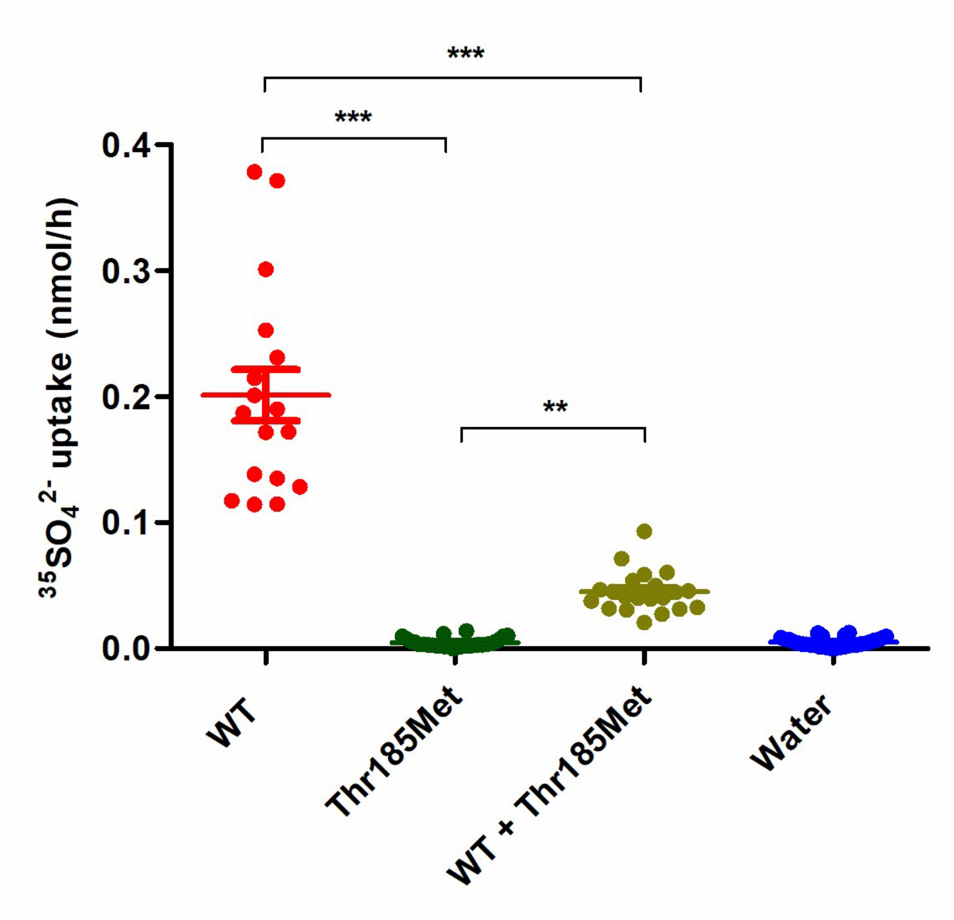

SLC26A1-mediated SO_4^{2-} uptake by oocytes previously water-injected or injected with 10 ng of cRNA encoding Wild-Type SLC26A1 (WT) or Thr185Met mutant SLC26A1. A dominant negative effect was assessed by co-injecting WT:Thr185Met mutant (1:1) with a total cRNA of 10 ng. The experiments were performed with oocytes from 3 different frogs (and 3 different cRNA preparations) with a total number of 17 (WT), 28 (Thr185Met), 21 (WT:Thr185Met), and 30 (water-injected). Error bars, means ± SEM, One-way ANOVA, Bonferroni multiple comparison test ***p <0.001, **p <0.01.

Supplementary References:

1. Bowling FG, Heussler HS, McWhinney A, and Dawson PA. Plasma and urinary sulfate determination in a cohort with autism. *Biochem Genet.* 2013;51(1-2):147-53.
2. Wu M, Heneghan JF, Vandorpe DH, Escobar LI, Wu BL, and Alper SL. Extracellular Cl(-) regulates human SO4 (2-)/anion exchanger SLC26A1 by altering pH sensitivity of anion transport. *Pflugers Arch.* 2016;468(8):1311-32.
